# Light Activated Disinfection in Root Canal Treatment—A Focused Review

**DOI:** 10.3390/dj6030031

**Published:** 2018-07-10

**Authors:** Islam A. Abdelaziz Ali, Prasanna Neelakantan

**Affiliations:** Discipline of Endodontology, Faculty of Dentistry, The University of Hong Kong, Hong Kong, China; u3004980@connect.hku.hk

**Keywords:** root canal, biofilm, light activated disinfection, photodynamic therapy, photosensitizers

## Abstract

Light activated disinfection (LAD) is a strategy for optimizing root canal disinfection by using a highly-selective, targeted killing of bacteria using a combination of photosensitizers and light. Over the past decade, numerous in vitro and clinical studies have been performed to demonstrate the effectiveness of this mode of root canal disinfection. While most studies offer an important understanding of the effectiveness of LAD on monospecies biofilms, few have offered credence to the fact that infections of the root canal system are mediated by polymicrobial biofilms. Hence, it is imperative to understand the effect of LAD on polymicrobial biofilms both in terms of microbial killing and the changes in the biofilm architecture. The aim of this review was to systematically review the literature to evaluate the effect of LAD on dual and multispecies biofilms and demonstrate the antibiofilm effect of LAD. Two databases (PubMed and Scopus) were searched to identify eligible studies using a combination of key words. These studies were reviewed to draw conclusions on the effect of LAD on dual and multi species biofilm and the antibiofilm effect of LAD. It was found that LAD alone may be unable to eradicate dual and multispecies biofilms, but it may enhance the effect of conventional canal debridement strategies. Novel formulations of photosensitizers with nanoparticles showed the potential to inhibit biofilm formation and/or disrupt the biofilm architecture.

## 1. Introduction

Root canals are cleaned and shaped to remove inflamed and/or necrotic pulp tissue, microbial biofilms, and microbial toxins, which prevents or allows healing of the periradicular tissues [1,2]. Cleaning and shaping (which for the purposes of this review will, hence, be collectively termed root canal debridement) is accomplished by using a combination of instruments and chemical adjuncts. Thus far, achieving sterility of the root canal system appears to be an intangible goal. That said, it remains unknown if such sterility is required for successful clinical outcomes. It is more important to reduce the microbial load to a specific threshold at which the body’s immune system can initiate healing [3]. Interestingly, this threshold has not been defined and, hence, clinical protocols must be designed to achieve as much microbial reduction as possible.

There are two main issues that mitigate “optimal” disinfection of the root canal system: (i) Organization of microbes into biofilm communities and (ii) the anatomical complexities of the root canal system, which result in hard-to-reach areas, such as accessory canals, isthmi, and ramifications. Furthermore, the complex dentin structure, with numerous dentinal tubules, serve as niche areas for microbes [3]. With regards to biofilms, these complex, organized microbial entities are at least 1000-fold more resistant to antimicrobial therapies than their planktonic counterparts [4]. The extracellular polymeric matrix of these biofilms serves as a diffusion barrier, thereby, preventing penetration of antimicrobial agents inside the biofilms to kill the microbes and further disrupting the biofilm architecture [5,6]. Of the chemical agents used as irrigating solutions in contemporary endodontics, only sodium hypochlorite (NaOCl) appears to be able to disrupt biofilms. Thus far, this antiseptic remains the gold standard as it is the only chemical adjunct that can dissolve pulp tissue, disrupt biofilms, and kill microbes in biofilms [7]. However, delivery of chemical adjuncts to the anatomical eccentricities is still a clinical challenge.

One of the main disadvantages of NaOCl is its non-specific interaction with organic matter. This includes microbial cells, biofilm matrix, pulp tissue, host cells, and dentinal collagen. Such non-specificity is a clinical problem in terms of cytotoxicity to the host tissues and proteolytic effects on root dentin collagen, resulting in weakening of the tooth [8]. This is further exemplified when sequential irrigating regimens of sodium hypochlorite and demineralizing agents are used. Over the past decade, substantial efforts have been focused on developing alternate irrigation strategies that are specific to the microbial cells with either added or no benefit to the host tissues, specifically dentinal collagen. The most promising approach in this context is the use of photosensitizers to achieve disinfection [9,10].

Light activated disinfection (LAD), also termed photodynamic therapy (PDT) or photoactivated disinfection, is based on three elements: The photosensitizer (a non-toxic dye), the target cell or tissue, and a low-intensity light source with a specific wave length [11]. On sensitization of the tissue with the photosensitizer (PS) and subsequent light exposure, singlet oxygen and oxygen radicals are released, resulting in the rupture of microbial cells. Internalization of the PS results in further damage to the microbial cellular biomolecules [12,13]. Since the introduction of LAD in endodontics, numerous studies have been carried out to demonstrate the effect of different photosensitizers on the root canal microbiota. However, the diversity of the results as compared to conventional irrigation strategies has resulted in a reluctance towards the adoption of this strategy in routine clinical practice. This variability in the results of these in vitro studies are mainly due to three main factors: (i) Study design—biofilms vs. planktonic cells; (ii) parameters of light activation; and (iii) differences in PSs used. It remains inconclusive if LAD is an alternative disinfection strategy or adjunct to conventional disinfection. Most studies have evaluated the effectiveness of LAD on monospecies biofilms. However, root canal infections in vivo are characterized by a diversity of microbial species enclosed in a self-produced matrix of different macromolecules i.e., multispecies biofilms. Furthermore, it is also imperative to assess the biofilm architecture after such a disinfection strategy.

Thus far, no review has summarized the effect of LAD on multispecies biofilms and the biofilm structure. The aim of this focused review was to systematically search the literature to identify and review the papers that evaluated the effect of LAD on multispecies biofilms (including dual species) and the antibiofilm effect of LAD.

## 2. Methods

Two databases (PubMed and Scopus) were searched systematically using a combination of key words (from January 1995 to April 2018) based on the PICO framework: Population: root canal biofilm, Intervention: Light activated disinfection, Comparison: root canal irrigants, Outcome: Antibiofilm effects. Keywords related to each of these terms were used and the search strategy was modified based on the database used. A sample search strategy (used for PubMed) has been shown (Table 1).

**Figure 1:**
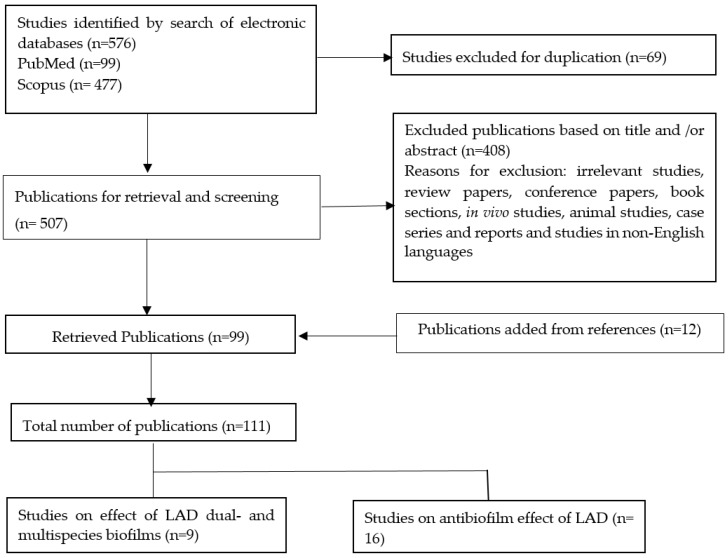
Flow diagram of the review search process and results.

The search strategy followed the PRISMA guidelines (Figure 1). Articles were included in this review if they evaluated the: (i) Effect of LAD on dual and multispecies biofilms (in vitro, ex vivo) or (ii) the antibiofilm effect of LAD.

## 3. Results

The initial electronic search revealed 576 articles (477 articles in Scopus and 99 articles in Pubmed). After exclusion of duplicates (69 articles), 507 articles were screened and, as a result, 409 articles were excluded based on the title and/or abstract. The reasons of exclusion were irrelevant studies, reviews, book sections, in vivo studies, animal studies, case reports, and studies in non-English languages. The relevant articles were screened to determine the in vitro studies that addressed the effect of LAD on dual- and multispecies biofilms, and the antibiofilm effect of LAD.

Nine papers evaluated the effect of LAD on dual and multispecies biofilms. Two of them investigated the effect of LAD on dual species biofilms, six on multispecies biofilms, and one on both dual and multispecies biofilms.

Considering the antibiofilm effect of LAD, sixteen studies were included. All of them were conducted on *E. faecalis* biofilms developed for 24 h–4 weeks. *Enterococcus faecalis* was the target microorganism as a monospecies biofilm in most of the studies. Two studies additionally included *Pseudomonas aeruginosa* as a target microorganism either as a monospecies biofilm [9,14] or in a mixed biofilm with *E. faecalis* [15]. One study included *Candida albicans* monospecies biofilm and in a mixed species biofilm of *Candida albicans* and *E. faecalis* [16], and one study included in situ developed multispecies biofilm [17].

Among the identified studies, three were found to demonstrate the antibiofilm effect of LAD on dual or multispecies biofilms [15,16,18].

### 3.1. Effect of LAD on Dual and Multispecies Biofilm

While most of the studies generated biofilms inside root canals in controlled laboratory conditions, three studies used a rather unconventional method. These methods are interesting as they are ex vivo designs, wherein one study [19] was conducted on extracted human teeth ex vivo with pulp necrosis and a periradicular lesion. This study was included because root canal species in the collected samples were partially characterized at the baseline, which revealed 39 species in endodontic infections (indicating a multispecies biofilm). In two other studies, plaque samples were collected form healthy volunteers from the premolar/molar region and bovine dentine discs fixed on an intraoral orthodontic appliance [17,20]. Of the three studies, one [19] was included while two [17,20] were excluded because the microbiome in the collected samples was not reported. The findings of the included studies have been summarized (Table 2).

Methylene blue was the PS used in five studies [15,19,21,24,25], while toluidine blue was used in three studies [16,22,23], and Rose Bengal was used in three studies either alone [16,18] or functionalized on cationic nanoparticles [18]. One study used Zn(II)chlorin e6 methyl ester and synthetic porphyrin [16].

### 3.2. Antibioiflm Effect of Light Activated Disinfection

In this section, the included articles were screened to identify the studies which addressed the antibiofilm effect of LAD. The criteria [26,27] to include the studies are:

- Inhibition or reduction of the biofilm formation of the target microorganisms in response to LAD; and/or
- changes in biofilm characteristics, such as thickness, biomass, biovolume, and biofilm architecture in response to LAD.

Based on the previous criteria, 16 studies were identified that evaluated the antibiofilm efficacy of LAD. Among the included studies, eight studies used methylene blue [9,14,15,17,28,29,30,31]. Rose Bengal was used in six studies [9,16,18,29,32,33]. Three studies used Indocyanine green [31,34,35], and one of them used Indocyanine green loaded on nano-graphene oxide [35]. Two studies reported the effect of chitosan-Rose Bengeal conjugate [9,32] and two other studies used Rose Bengal functionalized chitosan nanoparticles [18,33]. Two studies used curcumin [8,36], while synthetic tetracationic porphyrin and Zn(II)chlorin e6 methyl ester were used in one study [16].

In the selected studies, experiments were conducted on monospecies biofilms. One study involved mono- and dual-species biofilm [16], and one study on multispecies biofilms [18]. The selected studies in this section addressed the effect of LAD on established biofilms except two studies, which demonstrated the inhibition of biofilm formation by light activated disinfection [31,35]. Table 3 summarizes the general characteristics of these studies. The characteristics of the studies [15,16,18] are presented in Table 2.

## 4. Discussion

### 4.1. Effect of LAD on Dual and Multispecies Biofilm

Fimple et al. used methylene blue (MB) as a photosensitizer against four predominant bacterial species of infected root canals [21]. In this study, the photosensitizer was left unirradiated for 2.5 min between two episodes of light activation to allow oxygen diffusion into the oxygen deprived areas of infected root canals, since the presence of oxygen in the vicinity is necessary for the generation of cytotoxic singlet oxygen and reactive oxygen species upon photoactivation, which destroy bacterial cells [13,26]. Greater reduction of bacterial viability was observed when photoactivated MB was dissolved in PBS compared to that observed when it was dissolved in BHI. This was attributed to the presence of serum in the BHI, which affected the antibacterial performance of photoactivated MB. This highlighted the inhibitory effect of serum on the antibacterial mechanism of LAD as shown later by Shrestha and Kishen [27].

Root canal microorganisms of ex vivo root canal infections were sensitive to LAD as an adjunct to conventional chemomechanical debridement as shown by Ng et al. In this study, bacterial survival was observed when methylene blue was light activated after chemomechanical debridement. Viability of key endodontic pathogens was reduced after exposure to LAD. However, more bacteria were recovered when the dentinal shavings were collected compared to those recovered from flushing the root canals after root canal disinfection, indicating that currently used disinfection approaches, including LAD, were unable to eradicate bacteria from the anatomical eccentricities of the root canal system [19].

Schniffer et al. demonstrated that supplementary LAD enhanced the killing activity of sodium hypochlorite immediately after treatment. Interestingly, bacteria were able to repopulate when the microbial samples were taken two and four days after the initial treatment, indicating that these approaches were unable to prevent reinfection. This was explained by the survival of *E. faecalis* inside the dentinal tubules. Aerobic bacterial mixtures were eradicated immediately after chemocmechanical debridement and PDT treatment. However, this effect was abolished a few days after the treatment. In contrast, the anaerobic bacterial mixture was highly susceptible to NaOCl irrigation and NaOCl irrigation followed by LAD, with no significant difference between them. This effect was long lasting, and bacteria were eradicated four days after treatment [23].

In the same context, Hoedke et al. evaluated the effect of LAD as an adjunct to current root canal irrigation protocols. Reduction of bacterial load was evaluated immediately and five days after the treatment to simulate the situation of unobturated root canals encountered during retreatment. It was concluded that LAD is unable to eradicate intracanal bacterial to a satisfactory level without prior chemomechanical debridement. Bacteria were recovered a few days after saline-LAD combination treatment. In contrast, when LAD was applied after NaOCl and CHX irrigation, it enhanced bacterial reduction compared to non-photodisinfected root canals [25].

One of the most promising approaches in light activated disinfection has been functionalizing PS on nanoparticles for better penetration into the biofilms and microbial cells, as well as into the intricate anatomy of the radicular space. Shrestha and Kishen developed a mature multispecies biofilm model to simulate the in vivo root canal microflora of infected teeth. In this study, Rose Bengal functionalized chitosan nanoparticles (RBCnps) were compared to Rose Bengal photosensitizer alone. The results were promising in the favor of RBCnps in terms of dentin cleanliness and the disruption of the biofilm structure, as shown by SEM and CLSM, respectively. The study emphasized the positive impact of chitosan nanoparticles on the antibacterial properties of photoactivated Rose Bengal [18].

Despite the differences in the cell wall composition between gram-positive and gram-negative bacteria, 5.25% NaOCl followed by LAD was the most effective protocol against the tested microorganisms, according to the study by de Oliveira et al. in which multispecies biofilms of gram positive, gram-negative bacteria, and fungi were inoculated into root canals prepared by single file instrumentation technique [24]. It is worth mentioning that the multispecies biofilm was developed for 72 h, which could be sufficient for adhesion and biofilm formation, but not enough for the formation of a mature complex multilayered biofilm. The results might have been different if the biofilm had been allowed to grow for a longer period due to deposition of extracellular polymeric substance that protects individual cells against antimicrobial therapeutics. Furthermore, the thicker the biofilm, the lesser the penetration of photosensitizers into the deep layers of biofilms and, thereby, a lesser effect of LAD is expected.

More recently, Zn(II)chlorin e6 methyl ester, derived from natural sources, has been used as a photosensitizer against *E. faecalis* and *C. albicans* cells in a mixed species biofilm. While this PS was similar in its antimicrobial efficacy to Chlorhexidine and EDTA, it was significantly less effective than NaOCl. In the same study, the effect of LAD on the cellular morphology was examined using microscopy imaging. At the cellular level, display of the “ghost cells” feature of *E. faecalis* revealed the evacuation of intracellular contents with an intact cell wall as a result of photoactivation of Zn(II)e6Me, while *C. albicans* cells showed disruption of the cytoplasmic membrane, cell membrane invaginations, and presence of extracellular vesicles at the cell surface. This possibly showed that Zn(II)e6Me might possess microorganism specific inactivation mechanisms [16].

From these studies, it is apparent that that effect of LAD on dual and multispecies biofilms remains to be extensively studied. Furthermore, the identified studies show diversity in the constituent microorganisms that form the biofilms. Also, it is obvious that LAD with conventional PS, such as MB and RB alone, are unable to eradicate dual and multispecies biofilm. Light activated disinfection can, thus, enhance the antimicrobial efficacy of root canal irrigants and disinfection strategies. Some photosensitizers, like RBCSnps and a natural chlorophyll derived photosensitizer, are promising, and they can eradicate polymicrobial biofilms more effectively compared to traditional photosensitizers. However, more studies are required to evaluate their effects on different combinations of microorganisms in a polymicrobial biofilm.

### 4.2. Antibiofilm Effect of Light Activated Disinfection

#### 4.2.1. Methylene Blue

Eight studies used methylene blue and its formulations as photosensitizers. Methylene blue, in association with verapamil hydrochloride as an efflux pump inhibitor, was used in two studies [29,30]. Methylene blue dissolved in different solvents was used in two studies [14,28]. In one study, despite the finding that photoactivated MB and RB were not significantly different in their bacterial killing effect on 4-days biofilms, photoactivated MB induced greater reduction in biofilm thickness and killed more bacterial cells compared to photoactivated Rose Bengal, as shown by 3-dimensional imaging using confocal microscopy [29]. The inferior results of RB compared to MB were attributed to the repulsion between the negatively charged cell wall of *E. faecalis* cells (due to lipoteichoic acid residues) and the anionic Rose Bengal molecules. The biofilm disruption by photoactivated MB was evident as it reduced the biofilm covered surface, with relatively few cell aggregates and a reduced extracellular matrix [15]. In another study, light activated MB exhibited higher antibiofilm efficacy compared to Ca(OH)_2_ and polycationic chitosan nanoparticles at higher concentrations. Addition of an efflux pump inhibitor improved the antibiofilm effect of MB to a higher extent compared to the other two disinfection strategies as EPI enhanced the diffusion of MB into the biofilm matrix and, subsequently, increased the production of singlet oxygen, resulting in the disruption of the extracellular polymeric matrix [30].

Variations in the biofilm thickness and oxygen concentration across the multi-layered biofilm results in a less than optimal performance of LAD using PSs. To allow better penetration of the PS to enhance the antibiofilm action, several modifications have been attempted [14,28]. These modifications included either inclusion of an oxygen carrier and oxidizer with MB [28] or dissolving of MB in a mixture of glycerol, ethanol, and water or dual stage approach of LAD, which consists of dissolving MB in a glycerol, ethanol, and water mixture, followed by illumination in an oxygen carrier solution. The latter approach was more effective than 1% NaOCl in eradicating *E. faecalis* biofilms from deep dentine layers [28,37].

Chemical modifications of MB formulations were found to exert more substantial biofilm destruction, reduction of the biofilm thickness, and more bacterial killing than MB dissolved in water. The superior bactericidal effect of MIX-based MB photosensitizer was attributed to the longer life of the generated singlet oxygen, impairment of cell membrane integrity, and extensive damage of chromosomal DNA induced by MIX-based formulations compared to the water-based MB [38]. Perfluoro-decahydronaphthalene serves as an oxygen carrier, which facilitated light penetration during the irradiation phase and increased the rate of singlet oxygen production [28,39]. Hydrogen peroxide as an oxidizer disrupted the biofilm matrix and facilitated penetration of PS into the biofilm. Upon interaction with MB, hydroxyl radicals are generated, which then inactivate the bacterial growth.

#### 4.2.2. Chitosan Rose Bengal Conjugate

Rose Bengal as a photosensitizer did not completely eradicate the biofilm bacteria due to the electrostatic repulsion between the Rose Bengal molecules and the negatively charged bacterial cells and exopolysaccharides, as mentioned previously [9,29]. Conjugation of the Rose Bengal photosensitizer with natural polymers was, therefore, proposed to improve the overall effect of Rose Bengal mediated LAD against resistant root canal pathogens. Two of the selected articles used Rose Bengal-chitosan conjugate and compared it with conventional PSs [9,32] Chitosan is a natural polymer with antimicrobial properties. Its unique chemical nature allows functionalization with various antimicrobial agents [40,41].

Chitosan-RB conjugate (CSRB) was able to substantially reduce the biofilm thickness and disrupt the biofilm architecture of *E. faecalis* and *P. aeruginosa*, unlike methylene blue and RB alone, which exhibited a lesser disrupting effect on the biofilm structure [9]. In addition, the efficacy of CSRB on biofilm cells was enhanced by increasing the photosensitization time, which did not influence the killing effect of light activated MB and RB. Interestingly, *P. aeruginosa* biofilm cells were completely eliminated in response to light activated CSRB at a light dose higher than 40 J/cm^2^. These findings were attributed to the overall positive charge of CSRB, which ensured an intimate contact of CSRB to the bacterial cell wall and a higher uptake of photosensitizers by bacterial cells [32]. In addition, the hydrophilic nature of chitosan allows deep penetration of CSRB through the water rich extracellular polymeric substance. The CSRB was further modified by functionalization of Rose Bengal with chitosan nanoparticles (RBCnps). Nanoparticles per se are known for their favorable physicochemical properties, including ultra-small size, higher chemical reactivity, and larger surface area/mass ratio, which suggests its widespread use in targeted drug delivery [42,43]. RBCnps were evaluated for their antibiofilm activity against mono- and multispecies biofilm [18,33]. In one study, RBCnps achieved complete elimination of *E. faecalis* mono-species biofilm cells after light activation by fractionated dosage, compared to continuous exposure to different light doses. Fractionation allows the replenishment of molecular oxygen, adjacent to the irradiated cells, and generates singlet oxygen molecules constantly [33]. RBCnps demonstrated disruption of the biofilm architecture and reduced viable bacterial cells of multispecies biofilm compared to Rose Bengal, in which the biofilm structure was not affected and aggregates of viable cells were still present among the dead cells [18].

The success of RBCnps over conventional photosensitizers was confirmed when RBCnps was challenged with tissue inhibitors. It was found that RBCnps preincubated with pulp remnants- for 24 h eliminated bacterial load after photoactivation. In contrast, photoactivated MB and RB were not able to eradicate bacterial cells [44].

#### 4.2.3. Indocyanine Green (ICG) and Its Modifications

In two studies, unmodified ICG was used [31,34], while one used ICG loaded on nano-graphene oxide [35]. In clinical situations, photosensitizers might not reach the target microbial cells at their lethal concentrations mainly due to the limited accessibility of light to the infection site. Therefore, different sublethal concentrations of MB, Toluidine Blue (TB), and ICG were tested against *E. feacalis* biofilm formation [31]. Biofilm formation was significantly reduced when the three photosensitizers were used at concentrations equivalent to at least one quarter of their minimum inhibitory concentration (MIC) values. ICG maintained its ability to reduce biofilm formation when used at a concentration equivalent to one sixteenth of the MIC value. On the other hand, one quarter and one eighth MIC of MB and TB were the smallest concentrations that were able to reduce *E. faecalis* biofilm formation. The lack of inhibition of biofilm formation by the lower concentrations creates a caveat for LAD that proper concentrations of PS are imperative for its clinical effects.

Clinical protocols of washing the PS or not prior to light activation also appears to have an influence on the required concentration and, ultimately, the antimicrobial efficacy. Chinifoursh et al. demonstrated that higher ICG concentrations were required to exhibit effective biofilm inhibition if it was washed prior to light irradiation [34]. This finding should be considered in clinical situations in which tissue exudates escape into the canal space, thereby, diluting the concentration of photosensitizers, and attenuating the effect of LAD on root canal microorganisms. It is worth mentioning that the antibacterial effect of ICG is not only related to generation of reactive oxygen species upon the light activation. Indocyanine green is light activated by near infrared light unlike other photosensitizers that are activated by visible light. ICG can convert most of the near infrared laser into heat, which causes thermal injury to the adjacent bacterial cells [45].

Despite the promising results of ICG as a photosensitizer with an antibiofilm effect, it still has some limitations, including concentration-dependent aggregation, rapid degradation, limited photostability, and reduced interaction of anionic ICG with the negatively charged bacterial cell surface [18,46]. Therefore, a nanoparticle-based approach has been implemented to improve ICG delivery and interaction with target cells by loading of ICG on nanographene oxide. High surface area, functionalization potential, and cost-effective synthesis are favorable characteristics that favor its application as a platform of anticancer drugs, antimicrobials, and proteins [47]. Nanographene oxide (NGO) loaded ICG-based PDT demonstrated a higher antibiofilm effect compared to ICG-based PDT due to stabilizing effect of NGO, which increased the bioavailability of ICG and singlet oxygen production in biofilms [35].

#### 4.2.4. Curcumin

Curcumin is a natural polyphenolic compound extracted from plants sources that possess antimicrobial properties [48,49]. Additionally, photoactivated curcumin appears to be an effective treatment strategy for persistent root canal infections [50,51]. The impact of light activation on antibacterial and antibiofilm properties of curcumin is still controversial. Neelakantan et al. revealed that light activated curcumin eradicated more viable biofilm cells from superficial and deep layers of the biofilm compared to curcumin alone and ultrasonically activated curcumin [8]. This was attributed to the release of hydrogen peroxide, which kills bacterial cells. However, curcumin alone and ultrasonically activated curcumin decreased biofilm mass compared to light activated curcumin. According to Pileggi et al., curcumin had a slight effect on *E. faecalis* cell viability and light activation was necessary to reduce the viability of biofilm cells [51]. Also, the irradiation time of curcumin is another critical factor, since curcumin irradiated for five minutes caused the greatest microbial reduction compared to curcumin irradiated for ten minutes [50].

Devaraj et al. used curcumin in a light activated intracanal medicament formulation and left it in the root canals for 14 days. The superior results of light activated curcumin over other conventional medicaments at different depths was attributed to its ability to exert a lethal effect on bacterial cells without being in close contact with the bacterial cells’ surface unlike most of the photosensitizers, which requires close approximation to target cells to kill bacterial cells or disrupt biofilms [36].

Based on the reviewed studies, conventional formulations of photosensitizers, such as methylene blue (MB) and Rose Bengeal (RB), when used alone, are unable to induce substantial alterations in the biofilm structure and biofilm related characteristics, like biomass and thickness. Modified formulations, either by changing the dissolving media (methylene blue dissolved in mixture of glycerol, ethanol, and water) or conjugation of photosensitizers with nanoparticles (such as Rose Bengal functionalized chitosan nanoparticles), demonstrated a higher efficacy in eradicating biofilm cells and disrupting the biofilm architecture. Further studies are needed to fabricate Nanocarrier systems for PS and to test their effect on the biofilm structure and biofilm matrix components of mono and multispecies biofilms of microorganisms relevant to persistent root canal infections.

## 5. Conclusions

The polymicrobial nature and complex structure of root canal biofilms, as well as the hypoxic root canal environment, are the main factors that influence the antimicrobial efficacy of light activated disinfection. Conventional root canal debridement using sodium hypochlorite irrigant can be enhanced by light activation of photosensitizers. Functionalization of photosensitizers with nanoparticles (e.g., chitosan with Rose Bengal) and naturally derived photosensitizers (e.g., Zn(II)e6 methyl ester and curcumin) can affect dual-and multispecies biofilms, reduce biofilm formation, and exert substantial alteration in biofilm structure.

## Figures and Tables

**Table 1:** Key words used for search strategy in PubMed.

Search Builder	Words Used	Results
#1	“root canal” OR dentin OR biofilm	99,272
#2	photodynamic OR “photodynamic therapy” OR photosensitizers OR “light activated disinfection” OR “photo-activated disinfection” OR “photodynamic disinfection” OR “photodynamic therapy” AND endodontics OR “light activated disinfection” AND endodontics OR “light activated disinfection” AND “root canal” OR “photo-activated disinfection” AND “root canal” OR “photo-activated disinfection” AND endodontics	54,750
#3	root canal irrigants” OR “endodontic irrigants” OR “intracanal medicaments” OR “root canal antiseptics” OR “intracanal dressings” OR “sodium hypochlorite” OR hypochlorite OR chlorhexidine OR alexidine OR MTAD OR Qmix OR “calcium hydroxide” OR “double antibiotic paste” OR “triple antibiotic paste	26,078
#4	antibacterial OR antimicrobial OR antibiofilm	1,614,194
#5	#1 AND #2 AND #3 AND #4	99

#1: Population, #2: Intervention, #3: Comparison, #4: Outcome, #5: Combined search builder (results of search builders #1, #2, #3 and #4 are combined).

**Table 2:** General characteristics of included studies on dual- and multispecies biofilms.

Study	Biofilm Characteristics	Photosensitizer/Irradiation Parameters	Experimental Groups	Methods of Evaluation	Main Results
Fimple et al. [21]	Multispecies (72 h) Actinomyces israelii *Fusobacterium nucleatum* subspecies *nucleatum* Porphyromonas gingivalis Prevotella intermedia	MB (25 µg/mL) dissolved in BHI or PBS (with or without light activation) PIT: 10 min 665-nm diode laser PD: 10 mW/cm^2^ E: 30 J/cm^2^ Irradiation for 2.5 min, 2.5 min break, 2nd exposure for 2.5 min	No comparative groups. Only MB was tested	DNA probe analysis CFU CLSM	*Actinomyces israelii* was the most affected following LAD MB and light combination induced the highest reduction in bacterial viability Destruction of biofilm species with foci of viable cells inside dentinal tubules after LAD
Ng et al. [19]	Multispecies (39 species from teeth with necrotic pulp and associated periradicular radiolucencies)	MB (50 µg/mL) 665-nm diode laser PD: 100 mW/cm^2^ E: 30 J/cm^2^ Irradiation for 2.5 min, 2.5 min break, 2nd exposure for 2.5 min	6% NaOCl 6% NaOCl + supplementary LAD with MB	CFU and whole genomic probe assay	LAD after 6% NaOCl reduced bacterial survival and posttreatment detection levels compared to 6% NaOCl only
Garcez et al. [15]	Dual species (72 h) Enterococcus faecalis Pseudomonas aeruginosa	MB (60 µM dissolved in distilled water) PIT: 2 min 660-nm diode laser P: 40 mWE: 9.6 J Irradiation for 4 min	No comparative groups. Only MB was tested	SEM	Higher reduction of *E. faecalis* compared to *P. aeruginosa* Alterations of biofilm cells size and shape, cell rupture, drainage of intracellular contents Disruption of *E. faecalis* biofilm with reduction of bacterial cells aggregates and extracellular matrix
Muhammad et al. [22]	Multispecies (7 days) Enterococcus faecalis Porphyromonas gingivalis Streptococcus salivarius Prevotella intermedia	TB PIT: 1 min Aseptium Plus^®^ LED light Irradiation for 2 min. TB (15 µg/mL) PIT: 1 min 650-nm diode laser P: 60 mW Irradiation for 2 min	All root canals were disinfected with PUI using 17% EDTA and 2.6% NaOCl prior to LAD with TB	Microbiological sampling and culturing SEM	PUI using EDTA and NaOCl eliminated bacterial load completely unlike LED and diode laser treatments Clean canal walls after PUI using EDTA and NaOCl
Schiffner et al. [23]	Aerobic bacterial mixture (72 h) *Enterococcus faecalis* *Shewanella putrefaciens* Anaerobic bacterial mixture (72 h) ○ Actinomyces naeslundii ➢ Bifidobacterium adolescentis ➢ *Peptostreptococcus* sp. ➢ Eggerthella lenta	TB PIT: 2 min 632–644 nm red light. P: 200 mW. Irradiation for 1 min	0.9% NaCl 1.5% NaOCl 1.5% NaOCl + supplementary LAD with TB	CFU	LAD enhanced the bactericidal activity of NaOCl against aerobic bacteria mixture immediately after treatment Anaerobic bacteria mixture was very susceptible to NaOCl and NaOCl + PDT and was completely eradicated 4 days after treatment
Shrestha and Kishen [18]	Multispecies (21 days) Streptococcus oralis Prevotella intermedia Actinomyces naeslundii	RB (10 mmol/L) RBCnps (0.3 mg/mL) PIT: 15 min 540-nm fiber light E: 60 J/cm^2^	RB RBCnps	SEM CLSM	Clean dentine surface and open dentinal tubules after RBCnps treatment Dense bacterial aggregate after RB treatment RBCSnps reduced biofilm thickness, killed cells and disrupted the biofilms
De Oliveira et al. [24]	Multispecies (72 h) Enterococcus faecalis Pseudomonas aeruginosa Staphylococcus aureus Candida albicans	MB (15 µg/mL) PIT: 2 min 660-nm diode laser P: 100 mW E: 8 J/sample Irradiation for 90 s	1% NaOCl 1% NaOCl + supplementary LAD with MB 5.25% NaOCl 5.25% NaOCl + supplementary LAD with MB 0.85% saline 0.85% saline + supplementary LAD with MB	CFU	5.25% NaOCl + LAD was the most successful protocol in eradicating the inoculated species Saline + LAD and 1% NaOCl protocols were not effective against tested microorganisms
Diogo et al. [16]	Dual species (48 h) Enterococcous faecalis Candida albicans	RB PIT: 15 min 557-nm green LED light P: 42 mW/cm^2^ E: 3780 J/cm^2^ TB, TMPyP and Zn(II)e6Me PIT: 15 min 627-nm red LED light P:35 mW/cm^2^ E: 3150 J/cm^2^ Irradiation for 60 and 90 s	3% NaOCl 2% CHX1 7% EDTA RB TB TMPyp Zn(II)e6Me	Safranin red assay Microscopic imaging techniques *	Zn(II)e6Me reduced biofilm biomass more than other PSs, was comparable to CHX and EDTA and less effective than NaOCl. Extensive damage of microbial cells ultrastructure by Zn(II)e6Me
Hoedke et al. [25]	Multispecies (5 days) Enterococcus faecalis Streptococcus oralis Prevotella intermedia	Phenothiazine chloride (10 mg/mL) PIT: 60 s 660-nm diode laser PD:100 mW/m^2^ E: 2.4 J/root canal Irradiation for 60 s.	0.9%saline + PS 1% NaOCl + PS 1% NaOCl and 2% CHX + PS (all groups were either light activated or left unirradiated)	CFU	NaOCl + CHX+ LAD induced higher bacterial reduction compared to other treatment groups immediately and 5 days after treatment. Saline + LAD was effective only immediately after treatment

BHI: Brain Heart Infusion, CFU: Colony forming units, CLSM: Confocal Laser Scanning Microscope, CHX: Chlorhexidine, EDTA: Ethylene diamine tetra acetic acid, E: Energy, LAD: Light activated disinfection, LED: Light emitting diode, MB: Methylene blue, NaCl: Sodium chloride (saline), NaOCl: Sodium hypochlorite, P: Power, PD: Power density, PBS: Phosphate buffered saline, PIT: Preirradiation time, PS: Photosensitizer, PUI: Passive Ultrasonic Irrigation, RB: Rose Bengal, RBCnps: Rose Bengal functionalized chitosan nanoparticles, SEM: Scanning Electron Microscope, TB: Toluidine blue, TMPyP: Synthetic tetra cationic porphyrin, Zn(II)e6Me: Zn(II)chlorin e6 methyl ester. (*): Microscopic imaging techniques include light and transmission electron microscopes.

**Table 3:** General characteristics of studies on the antibiofilm effect of light activated disinfection (LAD).

Study	Biofilm Characteristics	Photosensitizer/Irradiation Parameters	Experimental Groups	Methods of Evaluation	Main Results
George and Kishen [28]	*Enterococcus faecalis* (7 days)	Water-based MB (100 µmol/L) Emulsion-based MB (*) PIT: 10 min 664-nm diode laser P: 30 mW E:31.84 J/cm^2^	Water-based MB Emulsion-based MB	CLSM	Emulsion-based MBreduced biofilm thickness and caused marked biofilm disruption compared to water-based MB
Kishen et al. [29]	*Enterococcus faecalis* (4 days, 2 weeks)	MB, MB + EPI, and RB (100 µM) PIT: 15 min. Non-coherent light (660-nm for MB, 540-nm for RB) P: 300–600 mW E: 10–40 J/cm^2^	MB MB + EPI RB	CFU assay of biofilm cells and biofilm derived cells (4 days biofilm) CLSM (2 weeks biofilm)	MB + EPI induced maximum reduction of bacterial cells compared to MB and RB LAD with MB produced greater reduction of biofilm thickness compared to RB
Upadya and Kishen [14]	*Enterococcus faecalis* *Pseudomonas aeruginosa* Monospecies biofilms (2 weeks)	Water-based MBMIX-based MB (**) MB in MIX and Emulsion combination PIT: 15 min. 660-nm non-coherent light P: 0.106 W E: 2–40 J/cm ^2^	Water-based MB MIX-based MB	CLSM	MB in MIX and emulsion combination was the most effective in disrupting the biofilm structure and killing biofilm cells More apparent damage of *E. faecalis* biofilm structure compared to *P. aeruginosa*
Upadya et al. [30]	*Enterococcus faecalis* (4 days)	MB, MB + EPI PIT: 15 min 660-nm non-coherent light P: 0.106 W E: 2–40 J/cm ^2^	Ca(OH)_2_ Chitosan NPs MB Ca(OH)_2_ with EPI Chitosan NPs with EPI MB + EPI	CFU assay of biofilm cells and biofilm derived cells	light activated MB was more effective than Ca(OH)_2_ and Chitosan Nps Effect of EPI was more significant on antibiofilm effect of MB than that of Ca(OH)_2_ and Chitosan Nps
Shrestha and Kishen [9]	*Enterococcus faecalis* *Pseudomonas aeruginosa* Monospecies biofilm (7 days)	MB (10 µM) RB (10 µM) CSRB (0.3 mg/mL) White light source (540-nm for RB and CSRB, 660-nm for MB) E: 20, 40 and 60 J/cm^2^ (PIT: 15 min.) 40 J/cm^2^ (PIT: 30 and 60 min.)	MB RB CSRB	CFU assay of biofilm cells CLSM	light activated CSRP induced higher antibiofilm effect on biofilms of both microorganisms compared to MB and RB CSRP was the most effective in reduction of viable cells, biofilm thickness and disruption of biofilm architecture MB and RB were unable to disrupt biofilms
Shrestha et al. [32]	*Enterococcus faecalis* (7 days)	RB (10 µM) CSRB (0.3 mg/mL) PIT: 15 min. 540-nm light E: 20, 40 and 60 J/cm^2^	RBCSRB	CFU assay of biofilm cells	CSRP induced a significantly higher LAD mediated bacterial killing compared to RB at 40 and 60 J/cm^2^
Shrestha et al. [33]	*Enterococcus faecalis* (21 days)	RB (10 µM) RBCnps (0.1 and 0.3 mg/mL) PIT: 15 min. 540-nm light P: 50 mW E: 20, 40, 60 and fractionated dosage of 10 and 20 J/cm^2^ twice	RB RBCnps	CFU assay of biofilm cells CLSM	Complete elimination of biofilm cells by RBCnps (0.3 mg/mL) and RB exposed to fractionated dosage of LAD Complete killing was not achieved at higher light doses regardless the PS used Superior bacterial killing and complete disruption of biofilm structure following light activated RBCnps
Neelakantan et al. [8]	*Enterococcus faecalis* (4 weeks)	Curcumin (2.5 mg/mL) 380–515 nm blue light E:1200 mW/cm^2^ Irradiation for 4 min	Saline 3% NaOCl 3% NaOCl with PUI 3% NaOCl with LAD Curcumin Curcumin with PUI Curcumin with light	CLSM	Light activated curcumin was able to achieve higher killing of biofilm cells compared to sodium hypochlorite irrigation Curcumin and ultrasonically activated curcumin reduced biofilm mass more than light activated curcumin
Chiniforush et al. [34]	*Enterococcus faecalis* (24 h)	ICG (3–2000 µg/mL) PIT: 5 min 808-nm diode laser P: 250 mW E: 39.06 J/cm^2^ Irradiation for 60 s	No comparative groups Only ICG was tested	CV assay	Non-washed ICG induced higher reduction in biofilm formation, development and higher rate of biofilm degradation compared to washed ICG
Deveraj et al. [36]	*Enterococcus faecalis* (4 weeks)	Curcumin (2.5 mg/mL of polyethylene glycol) 380–315 nm blue light P: 1200 mW/cm^2^ Irradiation for 4 min	TAP DAP 2% CHX gel Ca(OH)_2_ gel Curcumin	CLSM	Light activated curcumin and TAP reduced biofilm thickness, disrupted biofilm architecture and killed bacterial cells more than other medicaments
Pourhajibagher et al. [31]	*Enterococcus faecalis* (24 h)	Sublethal concentrations of TB, MB (6.2 µg/mL) and ICG (31.2 µg/mL) Diode laser: 660 nm (MB), 635 nm (TB) and 810 nm (ICG) P: 150 mW (MB), 220 mW (TBO) and 200 mW (ICG) E: 70.31 J/cm^2^ (MB) 103.12 J/cm^2^ (TBO) 15.62 J/cm^2^ (ICG)	TB MB ICG	CV assay SEM	ICG-sPDT inhibited biofilm formation more than TBO-, MB-sPDT Lower cell density and more irregular shaped cells were observed in ICG-sPDT treated biofilms
Akbari et al. [35]	*Enterococcus faecalis* (24 h)	ICG (1000 µg/mL) NGO-ICG (200 µg/mL) 810-nm diode laser P: 250 mW E: 31.2 J/cm^2^ Irradiation for 60 s	ICG NGO-ICG	CV assay	Photoactivated NGO-ICG reduced biofilm formation more than photoactivated ICG
Rosa et al. [17]	Multispecies biofilm developed intraorally (72 h)	0.01%MB. PIT: 1 min 650-nm red diode laser P: 100 mW Irradiation for 1 min	Saline Saline + PDT with MB 2.5% NaOCl 2.5% NaOCl + PDT with MB 2% CHX 2% CHX + PDT with MB	CLSM	LAD after NaOCl reduced biofilm biovolume

CV: Crystal Violet, Ca(OH)_2_: Calcium hydroxide, CSRB: Chitosan rose Bengal conjugate, DAP: Double antibiotic paste. EPI: Efflux pump inhibitor. ICG: Indocyanine green, NGO-ICG: Nano-graphene oxide loaded with Indocyanine green, sPDT: Sub-lethal doses of photodynamic therapy. TAP: Triple antibiotic paste. (*) Emulsion-based MB: MB in an emulsion mixture of perfluoro-decahydronaphthalene, H_2_O_2_, and triton X-100. (**) MIX-based MB: Methylene blue dissolved in a mixture of glycerol, ethanol, and water.
